# Hydrothermal Synthesis and Characterization of Ni-Al Montmorillonite-Like Phyllosilicates

**DOI:** 10.3390/nano3010048

**Published:** 2013-01-21

**Authors:** Marc X. Reinholdt, Jocelyne Brendlé, Marie-Hélène Tuilier, Serge Kaliaguine, Emmanuelle Ambroise

**Affiliations:** 1 Université de Poitiers, CNRS UMR 7285 IC2MP, ENSIP-Bât. B1, 1 Rue Marcel Doré, F-86022 Poitiers cedex, France; 2 Université de Haute Alsace (UHA), CNRS, Equipe Matériaux à Porosité Contrôlée (MPC), Institut de Science des Matériaux de Mulhouse (IS2M), LRC 7228, F-68093 Mulhouse, France; E-Mails: jocelyne.brendle@uha.fr (J.B.); emmanuelle.ambroise@uha.fr (E.A.); 3 Université de Haute Alsace (UHA), Laboratoire Physique et Mécanique Textile (LPMT), EA 4365 (conventionnée au CNRS), F-68093 Mulhouse, France; E-Mail: marie-helene.tuilier@uha.fr; 4 Département de Génie Chimique, Université Laval, 1065 ave. de la Médecine, Québec, QC G1V 0A6, Canada; E-Mail: serge.kaliaguine@gch.ulaval.ca

**Keywords:** clay, montmorillonite, synthesis, fluoride medium, solid-state NMR, EXAFS

## Abstract

This work describes the first hydrothermal synthesis in fluoride medium of Ni-Al montmorillonite-like phyllosilicates, in which the only metallic elements in the octahedral sheet are Ni and Al. X-ray diffraction , chemical analysis, thermogravimetric and differential thermal analysis, scanning electron microscopy and transmission electron microscopy confirm that the synthesized samples are montmorillonite-like phyllosilicates having the expected chemical composition. The specific surface areas of the samples are relatively large (>100 m^2^ g^−1^) compared to naturally occurring montmorillonites. ^29^Si and ^27^Al nuclear magnetic resonance (NMR) indicate substitutions of Al for Si in the tetrahedral sheet. ^19^F NMR and Ni *K*-edge extended X-ray absorption fine structure (EXAFS) local probes highlight a clustering of the metal elements and of the vacancies in the octahedral sheet of the samples. These Ni-Al phyllosilicates exhibit a higher local order than in previously synthesized Zn-Al phyllosilicates. Unlike natural montmorillonites, where the distribution of transition metal cations ensures a charge equilibrium allowing a stability of the framework, synthetic montmorillonites entail clustering and instability of the lattice when the content of divalent element in the octahedral sheet exceeds *ca.* 20%. Synthesis of Ni-Al montmorillonite-like phyllosilicates, was successfully achieved for the first time. These new synthetic materials may find potential applications as catalysts or as materials with magnetic, optical or staining properties.

## 1. Introduction

Clay minerals have been widely used by human societies since the early days of mankind [1,2,3]. Modern uses of clay minerals cover a large variety of domains like building materials, ceramics, absorbents, ion exchangers, cosmetic and pharmaceutical products, oil drilling, foundry and paper industries, wastes confinement (including nuclear wastes), pollutants removal, catalysis and polymer nanocomposite reinforcement [4,5,6,7,8,9]. Clay minerals consist of a variety of phyllosilicates, commonly observed on Earth, which can be divided into several groups depending on the layer type (1:1, 2:1 or 2:1:1), charge per formula unit and nature of structural elements and interlayer species [3,10]. These groups are: kaolin-serpentine (1:1 phyllosilicate), pyrophyllite-talc, smectite, vermiculite, mica (all of them being 2:1 phyllosilicates) and chlorite (2:1:1 phyllosilicate). Among these various groups, smectites are largely used because of their high adsorption (so important that they are designated as swelling clays), acidic and cation exchange properties [3,10]. Within the smectite family, montmorillonite clay is the most common and used one, mainly because of the existence of large deposits, yielding a very low cost raw material [11,12]. The ideal 2:1 layer structure of montmorillonite is constituted by a sheet of AlM(OH)_2_O_4_ octahedra (M being a divalent element, mostly Mg) sandwiched between two sheets of SiO_4_ tetrahedra [11]. The intrinsic isomorphic substitution of Al by divalent elements in the octahedral sheet and the frequent substitution of Si by Al in the tetrahedral sheets are at the origin of the structure’s negative charge. This charge is compensated by cations located in the gallery between the clay sheets. Montmorillonite contains two Al(M) elements in the centre of two out of three octahedra, the third one being vacant, making it a member of the dioctahedral smectite subgroup. Tetrahedral substitutions, heterogeneous elemental compositions, varying particle size, structural clustering and important amounts of impurities, limit the use of natural clay minerals for particular applications such as catalysis [12].

Thus, it becomes interesting to explore the synthesis of some attractive clay minerals, like montmorillonite, to develop pure products with well-known compositions, morphologies, particle sizes and structural element arrangements for specific applications. In this context, synthesis of montmorillonite was specifically explored with the aim of obtaining reference clay-like compounds [3,13,14,15,16]. The hydrothermal synthesis route remains the preferred method used by scientists working either in materials or geological sciences, because it is a versatile and easy to use method, which allows the attainment of well crystallized materials. Conditions of synthesis of pure montmorillonite remains relatively soft, with temperatures in the range 100–475 °C, pressures below 10 MPa (autogenously generated) and up to 120 MPa, and mildly acidic to strongly basic pH values (*ca.* 5 to 14). While the synthesis of pure Zn-Al or Mg-Fe montmorillonite-like minerals, containing only Zn and Al or Mg and Fe in the octahedral sheet, has been achieved [17,18], the synthesis of Mg-Al montmorillonite-like phases, containing only Mg and Al in the octahedral sheet, was only accomplished recently [13,14,15,16]. A first breakthrough was realized by using the fluoride route synthesis method [13,14], which allows syntheses over a large pH range from acidic (pH ≈ 2–3) to strongly basic (pH ≈ 13). Additionally, fluoride (F^−^) acts as a mineralizing agent together or with replacement of hydroxide (OH^−^). Another great advancement was performed more recently by a two-step method involving a first part in which an amorphous gel is prepared by basification, from pH ≈ 2 to 6 using NH_4_OH, of the chemical reagents mixture inducing the precipitation of the gel [15,16]. The second step of this method consists of hydrothermally treating the resulting amorphous gel by controlling both temperature and pressure of the synthesis to crystallize the montmorillonite-like mineral. Since this method of synthesis uses an organosilicon compound as the silicon source, i.e. tetraethylorthosilicate, it does not mimick well the natural crystallization processes, however it enables well crystallized low-charge clay-minerals to be obtained with a controlled chemical composition. 

On another aspect, natural clay minerals are known to be efficient acid catalysts due to their Brønsted and Lewis acidities [12,19]. These naturally occurring minerals are non-corrosive, low-cost materials, can be reused and thus the amount of wastes is limited. However, several structural and chemical heterogeneities and the presence of impurities restrict the use of these natural clays for some catalytic applications. As a consequence, the design of synthetic clay minerals becomes attractive with the aim of tailoring their chemical composition, cation exchange capacity, acidity or swelling properties. In recent years interest was particularly directed toward manipulating the nature and the amount of heteroatoms in the clay layers through isomorphous substitutions. Among these substituted new catalysts, Ni-phyllosilicates have been recently evaluated for the epoxidation of (Z)-cyclooctene and the oxidation of cyclohexanone in the presence of benzonitrile (Ni-saponite) [20], and for the CO_2_ reforming of methane (Ni-lizardite and Ni-talc) [21,22].

In this context, the first goal of our study was to demonstrate that the synthesis of Ni-Al montmorillonite-like phyllosilicates, containing only Ni and Al in the octahedral sheet, is possible. But the second essential objective was to thoroughly characterize the structures and evaluate the textural properties of the new synthetic minerals. Syntheses were performed following the fluoride route by adapting the method used to prepare Mg-Al or Zn-Al montmorillonite-like phyllosilicates [13,14]. Synthesized Ni-Al containing samples were characterized using X-ray diffraction (XRD), chemical analysis, scanning electron microscopy (SEM), transmission electron microscopy (TEM), thermogravimetric and differential thermal analysis (TGA-DTA), nitrogen adsorption-desorption experiments using the Brunauer Emmett and Teller method (BET), solid state magic angle spinning nuclear magnetic resonance (MAS-NMR) for the ^29^Si, ^27^Al and ^19^F nuclei and Ni *K*-edge extended X-ray absorption fine structure (Ni *K*-EXAFS) spectroscopies. This is the first report of the synthesis of Ni-Al montmorillonite-like clay minerals, containing only Ni and Al in the octahedral layer.

## 2. Results and Discussion

The synthetic compounds prepared in this work are fine green powders, the color of which is more intense as the Ni content increases.

### 2.1. X-Ray Diffraction

**Figure 1:**
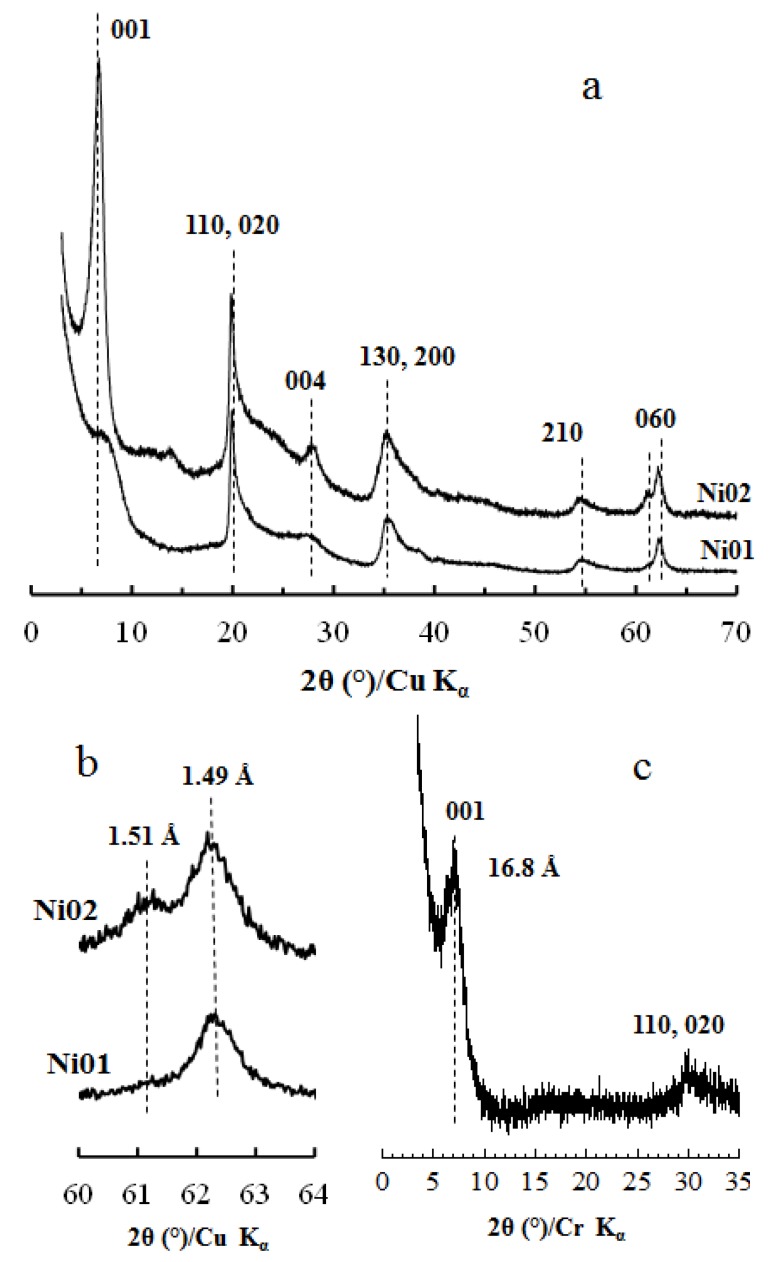
X-ray diffraction (XRD) patterns of synthetic Ni-Al phyllosilicates, samples Ni01 and Ni02: (**a**) whole diffractograms recorded at *P*/*P*_0_ = 0.80; (**b**) position of the (060) peak and (**c**) pattern of sample Ni02 (*x* = 0.2) exchanged with hexadecyltriethylammonium bromide.

XRD patterns of samples Ni01 and Ni02 exhibit *hk* bands and 00*l* reflections characteristic of phyllosilicates (Figure 1a). More specifically, the bands appearing at *ca.* 20, 35, 55 and 62° 2θ, can be indexed as the (02, 11), (13, 20), (15, 24, 31) and (06, 33) bands of a smectite mineral [11,12,23,24,25]. The number of octahedra occupied by metal elements defines the di- or trioctahedral character of the clay, *i.e.*, one or no vacancy out of three octahedra respectively. The position of the (06) band is related to this character, with associated *d*_060_ periodicity values of 1.49 and 1.52 Å respectively. Figure 1b displays the (060) bands of samples Ni01 and Ni02. The *d*_060_ periodicity of sample Ni01 is dominated by a peak centered at *ca.* 1.49 Å making it a solely dioctahedral mineral. Sample Ni02 exhibits two components, a main one at 1.49 Å and a secondary one at 1.51 Å, the latter demonstrating a partial trioctahedral character of the layers. The position of the (001) peak (Figure 1a), observed at *ca.* 12.7 and 13.2 Å for Ni01 and Ni02 samples respectively, gives the value of the interlayer distance and is typical of the smectite mineral family. The much broader (001) peak observed for Ni01 sample is characteristic of a reduced size of the coherent scattering domains perpendicular to the layer plane. To confirm these swelling properties, sample Ni02 was further subjected to hexadecyltrimethylammonium (HDTMA) intercalation. The intercalated hybrid-mineral shows a *d*_001_ spacing at *ca.* 16.8 Å (Figure 1c), which corresponds to values usually observed for a laterally intercalated monolayer of HDTMA in montmorillonite minerals [26,27,28,29].

### 2.2. Chemical Analysis

The two samples were analyzed for their chemical content (wt.%): Ni01 (Si: 27.40; Al: 9.00; Ni: 3.20; Na: 0.40; F: 0.47) and Ni02 (Si: 25.50; Al: 8.20; Ni: 7.50; Na: 0.40; F: 0.35). The amounts of Si, Al, Na and F are relatively similar in both samples, but the amount of Ni is more than doubled in sample Ni02 compared to sample Ni01, as expected from the chemical composition ot the initial reacting mixtures. Additionally, it seems that the Al content slightly decreases as the Ni content increases, as one would expect with a partial substitution of Al by Ni in the octahedral layer. This higher Ni content in the Ni02 sample corroborates the more intense color observed for that sample with respect to sample Ni01.

### 2.3. Scanning and Transmission Electron Microscopy

**Figure 2:**
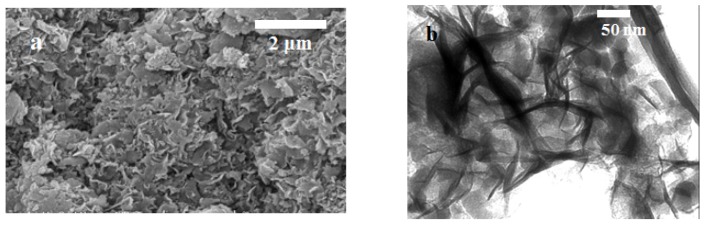
Representative (**a**) scanning electron microscopy (SEM) and (**b**) transmission electron microscopy (TEM) micrographs of our synthetic Ni-Al phyllosilicates.

Typical SEM and TEM pictures of samples Ni01 and Ni02 are shown on Figure 2. The synthetic inorganic compounds exhibit the well-known sand rose or cornflake morphology (Figure 2a), which is typically observed for natural clay minerals [30,31,32], especially for swelling phyllosilicates of the smectite family.

TEM micrographs of our samples (Figure 2b) show that the synthetic minerals seem to possess a layered structure characteristic of smectite clay minerals, either natural [27,33] or synthetic [15].

### 2.4. Thermal Analyses

**Figure 3:**
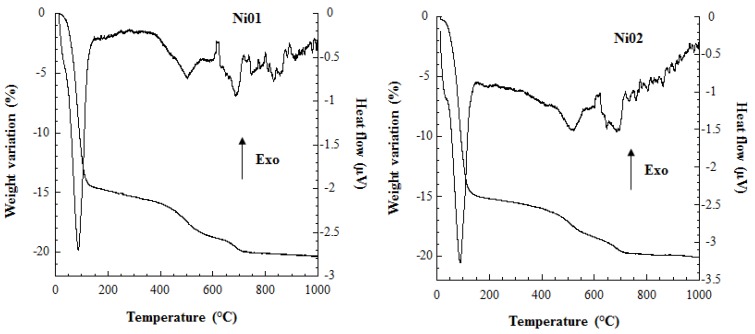
Thermogravimetric and differential thermal analysis (TGA-DTA) patterns of samples Ni01 and Ni02.

The thermogravimetric and thermal analysis curves of samples Ni01 and Ni02 are displayed in Figure 3.

The curves are very similar for both samples and exhibit a first main weight variation below 110 °C of *ca.* 14.4 and 15.4 wt.% for samples Ni01 and Ni02, respectively. This weight loss is associated with a large endothermic peak on the heat flow vs. temperature curve and they are both associated with clay mineral interlayer dehydration [34,35,36,37]. Both samples experience also two weight losses at *ca.* 500 and 680 °C, combined to endothermic peaks, which are of the order of 3.0–3.3 and 1.4–1.5 wt.%, respectively. These features are attributed to the dehydroxylation of the clay layer. The dehydroxylation appearing at the highest temperature is characteristic of montmorillonite clay minerals [34,35,36] and was also observed for a synthetic montmorillonite-like mineral, which was hydrothermally synthesized at higher pressure and temperature than in our case [15]. The dehydroxylation appearing at lower temperatures has already been observed in some natural montmorillonites [34,37], and is also a peculiar feature of our synthetic Mg-Al or Zn-Al montmorillonite-like minerals, containing only Mg and Al or Zn and Al in the octahedral layer [13,14]. These features correspond to two types of structural hydroxyls, assigned to *trans*- and *cis*-vacant sites, *i.e.*, the two occupied octahedra are positioned perpendicularly or parallel to the line formed by the hydroxyls in the structural plane, and associated with the lower and higher temperature dehydroxylation peaks respectively [35,36]. Thus, the presence of *cis-* and *trans-*vacant sites may arise from structurally different layers, *i.e.*, *cis* or *trans* 2:1 layers [35,36], or from a clustering of either the dioctahedral elements or the hydroxyl/fluorine groups or both during the synthesis process. Such possible clustering was observed in montmorillonite-like samples synthesized at low temperature and pressure and in slightly acidic and fluoric medium (see [13,14] and *vide infra*). As mentioned above montmorillonite-like minerals synthesized at higher temperature and pressure seem to contain only *cis-*vacant sites in their structure making them more homogeneous minerals. As a consequence of the previous discussion, the dehydroxylation observed at *ca.* 450–500 °C might be considered as a fingerprint of montmorillonite-like minerals synthesized in conditions similar as those presented here and in our previous studies [13,14]. Additionally, it is noteworthy that dehydroxylations of the Ni-Al montmorillonite-like minerals appear at temperatures slightly higher (*ca.* 20 to 40°) than those observed for the Mg-Al or Zn-Al synthetic montmorillonites [13,14], making the former slightly more thermally stable.

### 2.5. N_2_ Adsorption

The specific surface areas (SSA) of the samples are 115 and 134 m^2^ g^−1^ for Ni01 and Ni02, respectively. These values are higher than those usually observed for natural montmorillonites [38,39,40,41], though they are not unexpected since a synthetic Mg-Al montmorillonite showed a SSA of *ca.* 128 m^2^ g^−1^ [42]. However, this synthetic montmorillonite-like clay mineral was recently studied for its dissolution kinetics [43], and a purified sample exhibited a 104 m^2^ g^−1^ SSA. Thus the high SSA values observed for the Ni-Al montmorillonite-like synthetic minerals are probably due to the presence of an additional silica amorphous phase detected by ^29^Si NMR (*vide infra*). This assumption is sustained by the fact that the SSA value increases from sample Ni01 to Ni02, as the amount of silica seems to increase (*vide infra*).

### 2.6. Solid State Nuclear Magnetic Resonance

In order to see whether there is also Al for Si substituted in the tetrahedral sheet, ^27^Al and ^29^Si solid state MAS-NMR experiments were carried out.

**Figure 4:**
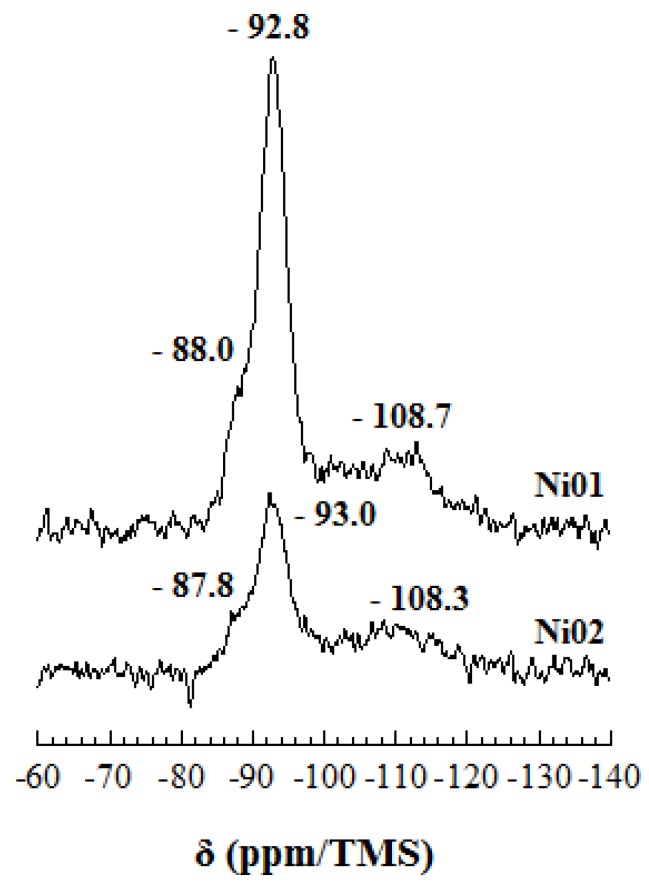
^29^Si nuclear magnetic resonance (MAS-NMR) spectra of samples Ni01 and Ni02.

The ^29^Si MAS-NMR spectra of samples Ni01 and Ni02 exhibit a main peak at *ca.*−93 ppm, a shoulder on its left side at *ca.*−88 ppm and a very broad, though relatively important, peak at *ca.*−108 ppm (Figure 4). These features can be readily assigned to the Si(0Al) environment [44,45,46,47], *i.e.*, Si surrounded by three Si in the tetrahedral sheet, the Si(1Al) environment [44,45,46,47], *i.e.*, Si surrounded by two Si and one Al in the tetrahedral sheet, and to amorphous silica [14], respectively. The Si(1Al) environment occurrence is characteristic of Al for Si substitutions in the tetrahedral sheet. A slight shielding of the Si(0Al) environment chemical shift, *ca.* 0.2 ppm, is observed between samples Ni01 and Ni02. This slide of the chemical shift to higher field values may be related to the partial trioctahedral character of Ni02 sample [46,47], in agreement with XRD observations (*vide supra*). The signal to noise ratio of the spectra is relatively low and probably due to the presence of amorphous silica. The presence of amorphous silica was also observed in the previously synthesized Mg-Al or Zn-Al montmorillonite-like phyllosilicates, but not to such an extent [14]. This demonstrates clearly that the conditions of synthesis of montmorillonite-like clay minerals, consequently their occurrence in nature, might be strongly affected by the type of metal cations present in the medium. Because of the low quality of the spectra, it was not possible to properly simulate them and gather more information.

**Figure 5:**
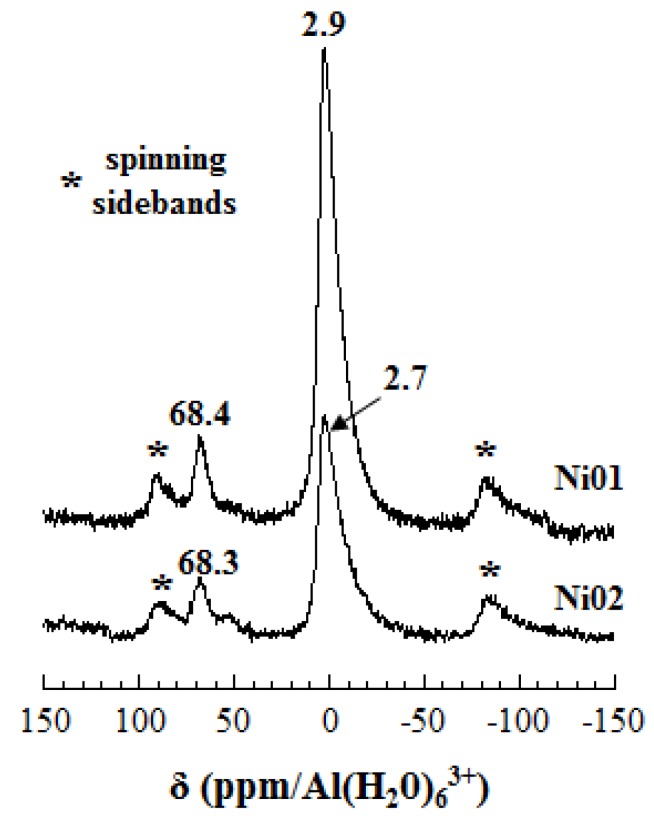
^27^Al MAS-NMR spectra of samples Ni01 and Ni02.

^27^Al MAS-NMR spectra of samples Ni01 and Ni02 (Figure 5) present a main asymmetric peak at *ca.* 3 ppm characteristic of the octahedral aluminum environment (Al_VI_) [44,46,48,49]. The resonances observed in the range 50–70 ppm are assigned to the tetrahedral aluminum environment (Al_IV_) [44,46,48,49]. The presence of these resonances confirms that there are both octahedral and tetrahedral substitutions in these synthetic montmorillonite-like Ni-Al phyllolicates. The Ni01 spectrum shows only one asymmetric peak at *ca.* 68 ppm, while the Ni02 spectrum shows two peaks, an asymmetric one at *ca.* 68 ppm and a smaller one at *ca.* 55 ppm. In the latter case, the existence of two different Al_IV_ resonances might be correlated to the clustering of the metallic elements in the octahedral sheet (*vide in*fra).

**Figure 6:**
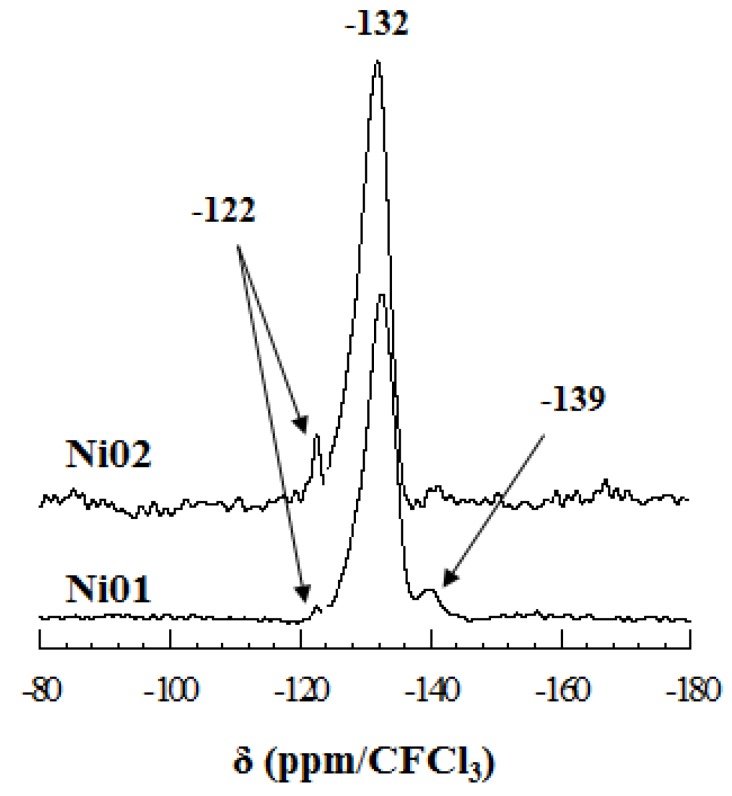
^19^F MAS-NMR spectra of sample Ni01 and Ni02.

**Figure 7:**
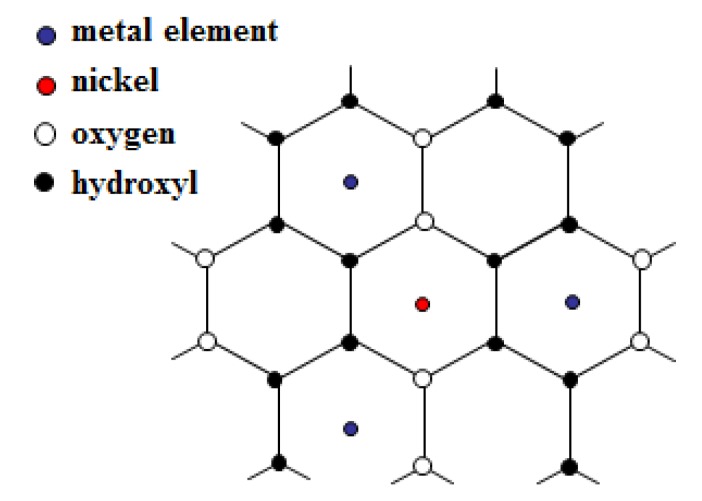
In plane structure of the octahedral layer.

Since fluorine atoms may have two or three octahedrally coordinated elements (Al, Ni) in their environment, fluorine was used as a local probe for the determination of the various tri-hexagonal sequences present in the octahedral sheet. Huve has shown that depending on the dioctahedral or the trioctahedral character a relationship can be established between the average of the electronegativity of the octahedral sheet elements (e_□_) and the theoretical ^19^F chemical shift (δ_19F_) [50]. In the case of dioctahedral clays the following relation can be used: δ_19F_ = 117 e_□_ − 247, whereas for trioctahedral clays it is the following one: δ_19F_ = 50 e_□_ − 238. Assuming the presence of Al-Al- , Al-Ni- or Ni-Ni-Ni tri-hexagonal sites, their theoretical chemical shifts should be −132, −121 and −150 ppm, respectively. However, the presence of a paramagnetic center, e.g., Ni, can cause (sometimes large) chemical shift changes, known as paramagnetic shifts, and in this case the experimental chemical shift can greatly differ from the theoretical one. ^19^F MAS-NMR spectra of samples Ni01 and Ni02 (Figure 6) present a main resonance at *ca.*−132 ppm, which is assigned to the Al-Al- environment [13,14,51], one fluorine surrounded by two aluminum and one vacancy in the three neighboring octahedra (Figure 7). Both spectra exhibit a peak at *ca.*−122 ppm, which may be assigned to an Al-Ni- environment [50]. Finally, both spectra show a third resonance at *ca.*−139 ppm, which might be assigned to the Ni-Ni-Ni environment [50], for which a chemical shift of about 10 ppm is observed with respect to the theoretical value. The chemical shift change observed in the case of the Ni-Ni-Ni environment and not for the Al-Ni- environment might be explained by the much stronger local concentration of Ni. The Ni-Ni-Ni environment is thus observed in both samples and especially in sample Ni01, which is a purely octahedral mineral according to XRD (*vide supra*). Since no such trioctahedral clusters are expected in sample Ni01, this is evidence of the clustering of Ni, but also of the vacancies and possibly of the fluorine in the octahedral sheet.

These NMR observations of the existence of a clustering of the metal elements in the octahedral layer support the results deduced from Ni *K*-edge EXAFS (*vide infra*).

### 2.7. Ni *K*-Edge Extended X-Ray Absorption Fine Structure

**Figure 8:**
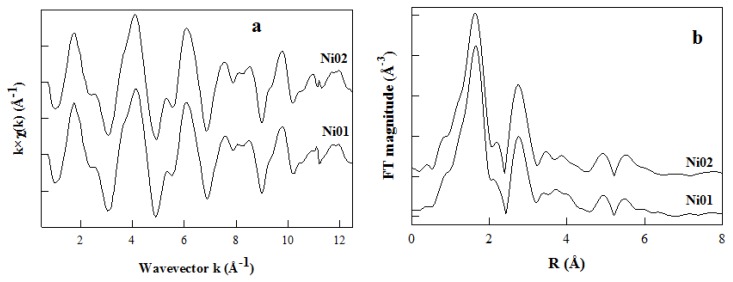
(**a**) Ni *K*-edge extended X-ray absorption fine structure (Ni *K*-EXAFS) spectra and (**b**) Fourier transformed (FT) magnitudes of the k^2^-weighted EXAFS data of the Ni01 and Ni02 samples.

Samples Ni01 and Ni02 were analyzed on synchrotron facilities to perform EXAFS measurements at the Ni *K*-edge (8333 eV). The experimental χ(k) spectra of the two samples and their Fourier transformed (FT) magnitudes are displayed in Figure 8a and b, respectively. One can easily observe that both sets of data are very similar, showing that the local environment of Ni atoms is much the same in both samples. The first and second main peaks on the FT magnitudes are assigned to the nearest neighbor (NN) and next nearest neighbor (NNN) shell contributions, respectively (Figure 8b). There are only tiny differences in the data sets and they are actually best noticed for values beyond 3 Å in the R space. As a consequence, differences between samples will only affect, slightly, the NNN shell contributions. The Ni NN shell contribution consists of oxygen atoms, and the NNN shell contains the Ni and Al atoms present in the surrounding octahedra and Si and Al nuclei present in the closest tetrahedra. While comparing this set of Ni *K*-edge data to those obtained at the Zn and Mg *K*-edges for montmorillonite-like phyllosilicates [13], one can easily observe that the NNN shell contributions of the FT spectra are more affected by the metallic elements content in the case of the Zn-Al or Mg-Al samples than in the case of the Ni-Al ones.

**Figure 9:**
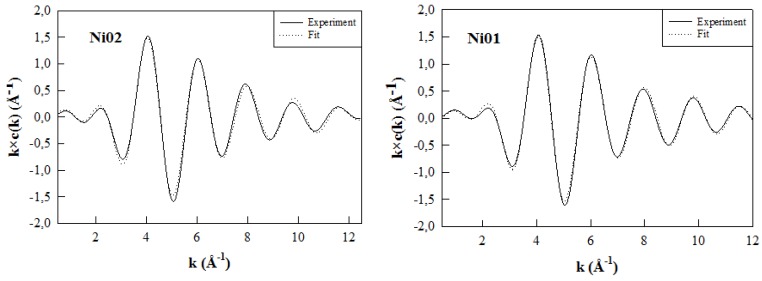
Best fits performed on the Fourier-filtered NN shell contribution of samples Ni01 and Ni02 using the parameters reported in Table 1.

The best simulations of the Fourier-filtered (FF) contribution of the NN peak for both Ni-Al samples are represented in Figure 9 and the structural parameters derived from these simulations are reported in Table 1. Ni nuclei have the same octahedral environment (six oxygen neighbors) in both Ni01 and Ni02 samples, as was observed previously for the synthetic montmorillonite-like phyllosilicates containing Zn and Al or Mg and Al (see samples Zn01, Zn02, Mg01 and Mg02 in Table 1) [13]. Additionally, one may see that the M-O distance is relatively close in the samples containing the same metallic element (M), meaning that it is independent of the amount contained in the sample. Though the M-O distance increases with the diameter of the M element, especially in the case of the two largest nuclei, Mg and Zn (Table 1; atomic radii: Ni^2+^− 0.69 Å, Zn^2+^− 0.74 Å, Mg^2+^− 0.72 Å). When comparing these M-O distances to the Al-O distance determined in Zn-Al or Mg-Al montmorillonites (*ca.* 1.93 Å) [52], or to the theoretical Mg-O and Al-O distances (2.15 and 1.91 Å, respectively) described for an ideal montmorillonite structure (Figure 1 in this reference) [53], one can deduce that the octahedron size increase induces enhanced distortions of the layer structure. 

**Figure 10:**
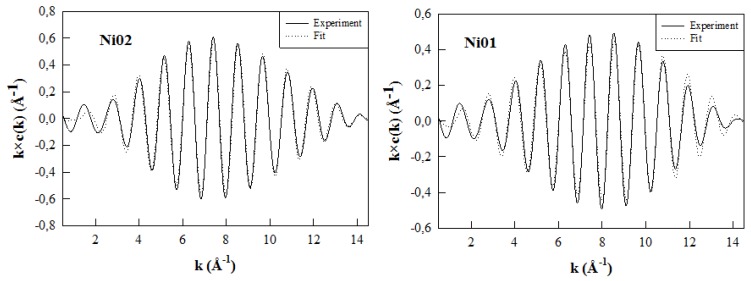
Best fits performed on the Fourier-filtered NNN shell contribution of samples Ni01 and Ni02 using the parameters and conditions reported in Table 1.

The best simulations of the FF contribution of the NNN peaks obtained for samples Ni01 and Ni02, with the number of M element fixed to four, are represented in Figure 10 and the structural parameters derived from these simulations are reported in Table 1, together with the parameters obtained for the simulations of NNN shell contributions at the Zn *K*-edge of samples Zn01 and Zn02 [13]. The mean Ni-Ni and Ni-Al interatomic distances slightly increase from 3.00 and 3.02 Å respectively in Ni01 to 3.03 and 3.05 Å in Ni02 (Table 1). Ni-Ni and Ni-Al distances in the octahedral sheet are very close and even Ni-Si distances between the elements located in the octahedral and tetrahedral sheets are similar. 

**Table 1:** Structural parameters derived from M *K*-edge EXAFS analysis of the NN and NNN shells for samples Ni01 and Ni02 (this work), samples Zn01, Zn02, Mg01 and Mg02 (references [13,52,54]).

	Sample	Shell	*N*	*D* (Å)	σ^2^ (Å)
NN shell	Ni01	Ni-O	6.0 ^a^	2.05 ± 0.02	0.005
	Ni02	Ni-O	6.0 ^a^	2.05 ± 0.02	0.005
	Zn01	Zn-O	5.9	2.08 ± 0.02	0.004
	Zn02	Zn-O	5.9	2.07 ± 0.02	0.004
		Al-O	6.0 ^a^	1.92 ± 0.02	0.008
	Mg01	Mg-O	6.0 ^a^	2.12 ± 0.02	0.006
	Mg02	Mg-O	6.0 ^a^	2.13 ± 0.02	0.007
		Al-O	6.0 ^a^	1.93 ± 0.02	0.006
NNN shell	Ni01	Ni-Ni	1.2 ^b^	3.00 ± 0.01	0.003
		Ni-Al	2.8 ^b^	3.02 ± 0.01	0.004
		Ni-Si	4.0 ^a^	3.20 ± 0.04	0.002
	Ni02	Ni-Ni	2.1 ^b^	3.03 ± 0.01	0.004
		Ni-Al	1.9 ^b^	3.05 ± 0.01	0.006
		Ni-Si	4.0 ^a^	3.21 ± 0.04	0.004
	Zn01	Zn-Zn	2.0 ^b^	3.11 ± 0.04	0.005
		Zn-Al	2.0 ^b^	2.98 ± 0.04	0.007
		Zn-Si	4.0 ^a^	3.17 ± 0.04	0.005
	Zn02	Zn-Zn	2.3 ^b^	3.11 ± 0.04	0.006
		Zn-Al	1.7 ^b^	2.97 ± 0.04	0.006
		Zn-Si	4.0 ^a^	3.25 ± 0.04	0.005

^a^ fixed parameter, ^b^ Sum of *N*_M-Al_ and *N*_M-M_ fixed to 4.0.

The proportion of Ni nuclei in the NNN shell increases with the Ni content, as one would expect (Table 1). Though these proportions, 1.2 and 2.1 in Ni01 and Ni02 respectively, are much larger than expected for a statistical distribution of the M elements in the octahedral sheet. The central Ni nucleus should indeed not witness any other Ni nucleus in its NNN shell in the case of Ni01 sample, and it should see less than one Ni nucleus in the case of the Ni02 sample, as in the case of Zn-containing phyllosilicates [13]. This is the evidence of the clustering of the M elements in the octahedral sheet of the Ni-Al montmorillonite-like phyllosilicates, containing only Ni and Al in this sheet, which confirms the ^19^F solid-state NMR observations. However, if there are similarities in the clustering of the M elements in both Ni-Al and Zn-Al containing samples, there are slight discrepancies in terms of the proportions of the M elements surrounding the central M nucleus, and there are significant differences concerning the values of the M-M distances. In fact, the proportion of Zn surrounding the central Zn nucleus seems to be slightly larger, for similar M elements content, than the proportion of Ni surrounding the central Ni nucleus (Table 1). This observation would lead to a greater clustering of the M elements in the case of the Zn-Al phyllosilicates, containing only Zn and Al in the octahedral sheet. Additionally, the Ni-Ni, Ni-Al and Ni-Si distances in the Ni-Al containing samples are close to the ideal M-M distance [13,55]. The Ni environment is less affected by the Ni content than the Zn environment is by the Zn content in the Zn-Al samples. The Zn-Zn distance is indeed longer than the Zn-Al distance (Table 1), consequently longer than the ideal M-M distance, and the Zn-Si distance increases with the Zn content of the samples (Table 1). These observations highlight the existence of distortions in the layer structure of the Zn-Al phyllosilicates, which may thus counterbalance the effect of the higher clustering of the elements compared to the Ni-Al phyllosilicates. Additionally, the Ni-Ni distances being significantly shorter than the Zn-Zn ones, also supports the idea of lesser clustering in Ni-Al montmorillonites than in Zn-Al montmorillonites. All these considerations explain the improved local order around Ni nuclei, which is sustained by lower Ni-Ni and Ni-Al Debye-Waller factors. As a consequence, the local environment of the Ni nucleus seems to be more organized than the local environment of the Zn nucleus in these kinds of montmorillonite-like phyllosilicates.

As a complement to the NNN shell study we would like to highlight that if the best simulations are obtained with a number of four M elements surrounding the central Ni atom, simulations using three M elements were also performed, but with less success (data not shown). Clearly, in the case of a statistical distribution of the M elements in the octahedral layer of a dioctahedral phyllosilicate, the number of hexagonal sites occupied around a given one is three out of six (Figure 7). However, if sample Ni01 is dioctahedral in nature, sample Ni02 is partially trioctahedral, as observed by XRD (*vide supra*). Thus, we conducted a first series of fit with three neighbors out of six around the central atom (dioctahedral character). Modifying the number of NNN elements surrounding the central Ni atom is possible since the FEFF input file used for the theoretical FEFFIT calculations (see experimental section below) contains coordinates of up to six surrounding M sites forming as many Ni-M pairs (M being Ni or Al). As expected, because of the results for Zn-Al montmorillonites [13], the simulations performed with three M neighbors did not fit well. Simulations with four neighbors were more successful, as explained above, which supports the existence of a clustering of the octahedral vacancies.

Al and Mg *K*-edge EXAFS analysis of synthetic montmorillonites containing Mg and Al in their octahedral sheet has revealed that the mean first shell coordination radii of Al and Mg are quite different: in fact the bond lengths have been found to be R_Al-O_ = 1.95 ± 0.03 Å and R_Mg-O_ = 2.11 ± 0.02 Å [52,54]. In the ideal montmorillonite structure, the smaller Al^3+^ cations (ionic radius: 0.54 Å) are located in M_2_ sites (R_Al-O_ = 1.95 Å) and the bigger Mg^2+^ cations (ionic radius: 0.72 Å) are located in M_1_ sites (R_Mg-O_ = 2.15 Å). Those values are very close to the Al-O and Mg-O bond lengths derived from Al and Mg *K*-edge EXAFS analysis performed on montmorillonites synthesized in fluoride medium [13,52]. Some of the octahedral sites are empty, in order to ensure a mean charge of two in the whole octahedral sheet.

In the Ni-Al montmorillonite, the mean Ni-O distance is found to be 2.05 Å. If Ni^2+^ divalent cations (ionic radius: 0.69) occupy the M_1_ sites, their local environment becomes distorted and differs from the ideal montmorillonite structure. The lattice distortion becomes more and more important as with increasing the Ni content. This interpretation is consistent with the increase of σ^2^ Debye-Waller factor in the Ni02 sample, with respect to the Ni01 sample (Table 1). Furthermore, the significant increase of the mean Ni-Ni and Ni-Al interatomic distances, from 3.00 and 3.02 Å respectively in Ni01 to 3.03 and 3.05 Å in Ni02 (Table 1), suggests that more adjoining octahedral sites are filled than in the ideal montmorillonite structure (R_M-M_ = 3.00 Å for both M_1_ and M_2_ sites). This result is consistent with the observation of Ni-Ni-Ni triads in ^19^F NMR together with the lack of Ni-Ni- environments. In fact, the octahedral sheet of natural montmorillonites contains transition metal cations either di- or trivalent, like Ni and Fe, and some vacancies, which ensures a suitable distribution of charges through the sheet. The presence of only Ni divalent transition metal cations in Ni-Al montmorillonite may induce a filling of empty octahedral sites as with increasing the Ni content. When divalent cations become in excess, they fill the empty octahedral sites in some parts of the octahedral sheet, leading to the splitting of the 060 reflection on XRD patterns, which is the fingerprint of di- and tri-octahedral character of the clay structure. Possible higher local charge within the octahedral layer, induced by the metal elements clustering, might be compensated by a lower local charge in the tetrahedral layer, which would be at the origin of the Al for Si substitutions in the tetrahedral layer.

## 3. Experimental Section

### 3.1. Synthesis

The samples presented in this work were hydrothermally synthesized in a fluoride medium using a 120 cm^3^ PTFE-lined stainless steel autoclave. The hydrogel composition was based on the following ideal montmorillonite-like phyllosilicate half-unit-cell formula: Na_2x_(Al_2(1__−*x*)_Ni_2*x*_□)Si_4_O_10_(OH)_2_, where *x* equals 0.10 or 0.20. Reagents were mixed in the following order: deionized water, hydrofluoric acid (HF, 40%; BDH, diluted to 5%), Na (sodium acetate, CH_3_COONa, 99%; Fluka), Ni (nickel acetate, Ni(CH_3_COO)_2_ 4H_2_O, 98%; Fluka), Al (pseudo-boehmite, Al_2_O_3_, 75%–78%, Pural SB1; Condea) and Si (SiO_2_, 99.5%, Aerosil 130; Degussa) sources to form hydrogels with the following composition 1·SiO_2_ : (1 − 2*x*)/4·Al_2_O_3_ : *x*/2·NiO : *x*/4·Na_2_O : 0.05·HF : 96·H_2_O. The amounts of deionized water, hydrofluoric acid and silicon oxide were the same for each synthesis. Hydrogels were matured for 2 h at room temperature and then autoclaved under autogenous pressure for 72 h at 493 K. After reaction, autoclaves were quenched to room temperature under a stream of water. The initial and final pH values were 5.5 (prior to autoclaving) and 3.5–4.0 (end of crystallization) respectively. Final products were thoroughly washed by filtration using deionized water and dried at 333 K overnight. Samples were then ground to a fine powder and placed under a relative humidity of *P*/*P*_0_ = 0.80, by using a saturated solution of ammonium chloride. Samples were labeled as Ni01 and Ni02 for *x* equals 0.10 and 0.20 respectively. Some Ni02 sample was ion-exchanged with hexadecyltrimethylammonium bromide (HDTMABr) (98%, Avocado) to probe the swelling properties of the sample.

### 3.2. Analytical Methods

#### 3.2.1. X-Ray Powder Diffraction (XRD)

The X-ray powder diffraction patterns were recorded on two different diffractometers. The first instrument was a PANalytical X’pert Pro diffractometer with fixed slits using Cu-K_α_ radiation (λ = 1.5418 Å) and θ–2θ configuration. Before analyses, powder samples were finely ground and pressed in a stainless steel sample holder. Data were recorded from 3 to 70° 2θ with a step size of 0.02°/point and a rate of 0.6° min^−1^ at a voltage of 35 kV and a current intensity of 55 mA. The second diffractometer employing Cr-K_α_ radiation with fixed divergence slits, Philips PW1100, was used to record more precisely the d-spacing (*d*_001_) of the Ni02 sample intercalated with hexadecyltrimethyl ammonium cations (HDTMA). Patterns were recorded from 1 to 35° 2θ with a step size of 0.02°/point and a rate of 0.2° min^−1^ at a voltage of 35 kV and a current intensity of 50 mA. In both cases diffractograms were performed on powders which were only pressed on sample holders. Information about the phase and *d*_001_ spacing was obtained with the APD 1700 software (Philips).

#### 3.2.2. Chemical Analysis

A part of each sample was Na-saturated to insure that only Na^+^ cations balanced the layer charge. Elemental analysis of the samples was performed by the Service Central d’Analyse of the Centre National de la Recherche Scientifique (Vernaison, France) for the following elements: Ni, Al, Si, and F. After mineralization, metal elements and fluorine contents were analyzed using inductively coupled plasma mass spectrometry.

#### 3.2.3. Scanning Electron Microscopy (SEM)

Starting materials were examined by SEM with a Philips XL30 microscope in order to determine their morphology. Prior to analysis, a few milligrams of powder was dispersed in ethanol and a few droplets of the suspension were left to evaporate on a holder covered with graphite. Samples were then gold-plated by sputtering under vacuum.

#### 3.2.4. Transmission Electron Microscopy (TEM)

Samples were examined by TEM using a Jeol JEM 1230 microscope equipped with a LaB_6_ filament and working at an excitation voltage of 80 kV. Prior to analysis, a few milligrams of sample was dispersed in methanol and a few droplets of the suspension were left to evaporate on a tiny nickel grid covered with a Formvar film.

#### 3.2.5. Thermogravimetric and Differential Thermal Analysis (TGA-DTA)

Measurements were carried out from room temperature to 1273 K at a heating rate of 5 K min^−1^ under a mixture of nitrogen and oxygen gas (1 and 0.5 bars, respectively) using a Setaram Labsys apparatus. Typically about 30 to 45 mg of powder previously equilibrated at a controlled humidity of *P*/*P*_0_ = 0.80 during 48 h was introduced in an alumina crucible to perform the measurement.

#### 3.2.6. Nitrogen Adsorption-Desorption Manometry Experiments (BET)

Nitrogen adsorption-desorption experiments were performed at the temperature of liquid nitrogen (77 K) using a Coulter Omnisorp 100 gas analyzer. The specific surface area was deduced from the most linear portion of the BET (Brunauer Emmett and Teller) plot (*P*/*P*_0_ = 0.006–0.10). The micropore volume was determined using the t-plot method. Samples were pretreated at 573 K under vacuum over 4 h prior to the analysis.

#### 3.2.7. Magic Angle Spinning Nuclear Magnetic Resonance (MAS-NMR)

The ^29^Si MAS-NMR spectra were recorded using a Bruker® MSL-300 spectrometer at 59.63 MHz, using a 7 mm MAS probe, 2 *μ*s excitation pulses (π/2 pulse width of 4 *μ*s) and 95 s recycle time. Spectra were recorded by accumulating 600 scans at a spinning rate of 4 kHz. Chemical shifts of Si were referenced to tetramethylsilane (TMS) using a secondary standard of trimethylsilylester of cubic octameric silicate (Q8M8) at -109.7 ppm (the more shielded signal). The ^27^Al MAS-NMR spectra were recorded on a Bruker® DSX-400 at 104.27 MHz, using a 4 mm MAS probe, 0.7 *μ*s excitation pulses (π/2 pulse width of 9 *μ*s for an aqueous solution) and 1 s recycle time. Spectra were recorded by accumulating 1200 scans at a spinning rate of 12 kHz. Chemical shifts of Al were referenced to a 1 M Al(NO_3_)_3_ aqueous solution with a chemical shift of 0 ppm. The ^19^F MAS-NMR spectra were obtained on the DSX-400 spectrometer at 379.23 MHz, using the 4 mm MAS probe, with a Hahn echo pulse sequence (π/2 pulse − τ − π pulse − τ − acquisition), 25–30 kHz spinning speed, π/2 pulse width of 4 *μ*s and 10 s recycle time. The value of τ was synchronized to a rotation period of the spinner. Spectra were recorded by accumulating 612 scans and the chemical shifts of F were referenced to octadecasil with a chemical shift of 0 ppm. After their collection, free induction decay (FID) signals were treated following a standard procedure using the WIN-NMR software (Bruker®, Bruker Biospin GmbH: Rheinstetten, Germany, 2001 ). The FID signals were smoothed in order to reduce the background noise and the base-line was also corrected if necessary. The ^29^Si MAS-NMR spectra were simulated with the WINFIT-2002 software using standard procedure [56].

#### 3.2.8. Ni *K*-Edge Extended X-Ray Absorption Fine Structure (Ni *K*-EXAFS)

The X-ray absorption experiments were performed at the Laboratoire pour l’Utilisation du Rayonnement Electromagnétique (LURE, Orsay, France). The Ni01 and Ni02 samples were mixed with cellulose (0.131 g of Ni01 and 0.77 g of Ni02 with 0.012 and 0.050 g of cellulose respectively) and pressed (1 T) in the form of pellets (less than 1 mm thick), which were maintained between two Kapton windows. Measurements were performed on the D21 beam line of the DCI storage ring. The X-rays were monochromatized by an Si(311) two-crystal spectrometer. The X-ray absorption spectra were recorded at the Ni *K*-edge (8333 eV) from 8200 to 9300 eV (1 eV step; 2 s^−1^ per point) in transmission mode at 20 K.

The raw data were processed following standard procedure by using the ATHENA routine (Bruce Ravel, The University of Chicago) [57], which works under the IFEFFIT interactive program for X-ray Absorption Spectroscopy (XAS) analysis (Matthew Newville, The University of Chicago, Chicago, IL, USA) [58]. First the pre-edge and post-edge backgrounds were removed from the raw data and the obtained absorption spectra normalized. The E_0_ origin of the kinetic energies of the photoelectron was taken at 8339 eV for all samples. After conversion to k-space (k being the wave vector of the photoelectron scattering on the neighboring atoms), the spectra were k1-weighted and Fourier-transformed (FT) between 2.0 and 13.8 Å^−1^ using a Kaiser-Bessel apodization window. 

The nearest neighbours (NN) and next-nearest neighbours (NNN) peaks in the Fourier transforms (FT) were assigned to the oxygen and metal backscatterers. The FT and Fourier-filtered (FF) contributions of oxygen NN and metallic NNN were simulated using the ARTEMIS routine [57] in order to derive the structural parameters: the interatomic distances *R*, coordination numbers *N* and Debye-Waller (DW) parameters σ. Montmorillonite structure possess two octahedral sites (M_1_ and M_2_) which are occupied by metallic cations, the mean M-O first distances being 2.15 and 1.91 Å for M_1_ and M_2_ sites, respectively [54]. Thus, the model was carried out by running FEFF-6 [59] with an input file constituted by the coordinates of the first O shell atoms surrounding an M_2_ octahedral site (arbitrarily chosen as the central atom), and then of the M_1_ and M_2_ octahedral sites and of the Si(Al) atoms present in the tetrahedral sheet for the second atomic shell. A Ni occupation level introduced in the *ab-initio* calculations was allowed to vary as other fitting parameters, and finely adjusted step by step.

## 4. Conclusions

In this work, synthesis of Ni-Al 2:1 phyllosilicates, containing only Ni and Al in their octahedral sheet, was performed under hydrothermal conditions using the fluoride medium route. The obtained materials are pale green powders exhibiting a layer structure and a sand rose morphology, as shown by TEM and SEM respectively, which is characteristic of montmorillonite minerals. The XRD and thermal analysis data exhibit characteristic features of clay minerals and more specifically to montmorillonite-like phyllosilicates. XRD also show that the sample containing the highest amount of Ni (Ni02) possesses a partial trioctahedral character. ^29^Si and ^27^Al NMR show the existence of Al for Si substituted in the tetrahedral sheet. ^29^Si NMR demonstrates the presence of amorphous silica in the samples, especially in sample Ni02. Chemical analysis confirms that the Ni content is somehow doubled from sample Ni01 to Ni02, as expected, but no chemical formula could be determined because of the presence of an amorphous silica phase. The samples have relatively large specific surface areas (>100 m^2^ g^−1^), though a non-negligible proportion of it comes from the amorphous phase. ^19^F NMR highlights a clustering of the metal elements and of the vacancies in the octahedral sheet of the samples. A Ni *K*-edge EXAFS study enabled the attainment in both samples of the Ni-M distances and the proportion of M elements (Ni or Al) surrounding the Ni nuclei. The analysis of these EXAFS data confirms unambiguously the clustering of both the M elements and the vacancies in the octahedral sheet, as observed previously in Zn-Al or Mg-Al montmorillonite-like phyllosilicates, containing only Zn and Al or Mg and Al in the octahedral sheet. The comparison of Ni and Zn *K*-edges results highlights a more important clustering of the M elements in the Zn-Al phyllosilicates than in the Ni-Al containing ones. Additionally, the Ni-Al phyllosilicates exhibit a higher local ordering around Ni. In natural montmorillonites, the distribution of di- and trivalent transition metal elements ensures the charge equilibrium over the whole octahedral sheet and allows a certain stability of the framework. Even if local defects lead to a trioctahedral character, the whole clay structure is preserved. Unlike natural montmorillonites, the synthetic montmorillonites were prepared from one sort of metallic divalent element, Mg^2+^, Zn^2+^, or Ni^2+^ in the present study. Their clustering in some part of the sheet results from the local loss of charge balance and leads not only to a trioctahedral character but also to severe distortions and instability of the structure when their content exceeds *ca.* 20%, as was observed in our previous study [13] and confirmed in the present work. Such charge and/or structural distortion defects may be compensated by Al for Si substitutions in the tetrahedral layer. The first goal of our study, the synthesis of montmorillonite-like Ni-Al phyllosilicates, containing solely Ni and Al in their octahedral sheet, was successfully achieved. This unprecedented success opens interesting perspectives on the synthesis of clay-like compounds containing various other transition metals in their structure. These new synthetic materials are presently being evaluated for their catalytic properties.
